# Causal relationship between air pollution and rheumatoid arthritis: A two-sample Mendelian randomization study

**DOI:** 10.1097/MD.0000000000042901

**Published:** 2025-06-20

**Authors:** Chengrui Yang, Zhiguo Du, Jing Ma, Lele Guo, Huidong Zhang, Yu Tian, Jianyong Zhao

**Affiliations:** a Cangzhou Hospital of Integrated Chinese and Western Medicine, Hebei Key Laboratory of Integrated Traditional and Western Medicine in Osteoarthrosis Research, Cangzhou, Hebei Province, China.

**Keywords:** air pollution, causality, environmental epidemiology, Mendelian randomization, rheumatoid arthritis

## Abstract

Recent studies have highlighted potential links between air pollution and autoimmune diseases. However, the causal relationship between air pollution (including PM2.5, PM10, nitrogen dioxide, and nitrogen oxides) and rheumatoid arthritis (RA) remains unclear. To address this, we performed a two-sample Mendelian randomization (MR) analysis to assess the causal effects of air pollution on RA. Genome-wide association study data for air pollution metrics were utilized as exposures, while RA outcome data were sourced from European cohorts. Inverse variance weighting served as the primary method for causal estimation, supplemented by sensitivity analyses including weighted median, MR-Egger, weighted mode, and Bayesian weighted MR. All analyses were conducted using R software. Results from the inverse variance weighting method indicated no significant causal association between PM2.5 (odds ratio [OR] = 0.71, *P* = .183, 95% confidence interval [CI]: 0.27–1.91), PM10 (OR = 0.657, *P* = .143, 95% CI: 0.374–1.154), nitrogen dioxide (OR = 0.482, *P* = .163, 95% CI: 0.173–1.343), or nitrogen oxides (OR = 0.868, *P* = .782, 95% CI: 0.317–2.373), and RA risk. Sensitivity analyses confirmed robustness with no evidence of pleiotropy or heterogeneity (*P* > .05). This study provides the first MR evidence suggesting that air pollution may not exert a genetically predicted causal effect on RA, offering critical insights for public health strategies and future research on environmental triggers of autoimmune diseases.

## 1. Introduction

Rheumatoid arthritis (RA) is a chronic autoimmune disorder that affects millions globally, characterized by joint inflammation, increased bone resorption, and decreased bone formation, ultimately resulting in significant bone loss. This condition primarily targets the small joints of the hands and feet and is associated with various systemic complications, including cardiovascular disease, pulmonary disorders, and increased susceptibility to infections. Such complications severely impair patients’ quality of life, leading to reduced physical activity and heightened emotional distress.^[1,2]^ The pathogenesis of RA is multifaceted, involving complex interactions between genetic susceptibility and environmental triggers.^[3]^ With the acceleration of industrialization and urbanization, air pollution may emerge as a potential risk factor for the development and exacerbation of RA.

Air pollution adversely impacts health through several mechanisms, particularly via inflammation and oxidative stress. Fine particulate matter (PM), including PM2.5 and PM0.1, along with ozone, nitrogen oxides (NO), and certain transition metals, serves as potent oxidants that generate reactive oxygen species. Extensive research has explored the relationships between these pollutants and various health outcomes. While some studies indicate a plausible link between air pollution and RA, traditional observational studies may yield biased results due to confounding factors and reverse causation. Additionally, experimental studies often face challenges related to the concentration and duration of air pollution exposure, as well as small sample sizes, which can compromise the accuracy of correlational evidence.^[4–7]^

The incidence of RA is closely related to various factors, including human leukocyte antigen genes, gender, age, and socioeconomic status. Although the association between inhalable pollutants, such as cigarette smoke, asbestos, and silica, and RA has been widely confirmed, the specific relationship between environmental air pollution and RA requires further investigation.^[8–10]^ Several studies and systematic reviews have explored the interactions between air pollution, RA, and other autoimmune diseases; however, existing data remain limited and inconsistent. For instance, a review by Essouma and Noubiap found an association between exposure to traffic pollution and an increased risk of RA.^[11]^ Di et al reported that although a significant positive correlation between PM10, carbon monoxide, and NO and RA could not be confirmed, PM2.5 may show a slight negative correlation. Additionally, living near high-traffic areas is also associated with an increased risk of RA.^[12,13]^ Some studies indicate a lack of consistent evidence supporting the association between exposure to NO_2_, PM2.5, and PM10 and RA risk.^[14–16]^ Therefore, a comprehensive investigation of the relationship between air pollutants and RA is crucial for understanding the environmental factors that affect this disease and its clinical significance.

In epidemiological research, Mendelian randomization (MR) serves as a valuable tool for assessing causal relationships between genetically predicted exposures and health outcomes. This method relies on the random transmission of genetic information at conception, effectively minimizing genetic confounding influences. By reducing potential confounding factors and the likelihood of reverse causation,^[17,18]^ MR has emerged as an effective alternative to randomized controlled trials in scenarios where cost and feasibility present significant challenges.

Our study aims to investigate the genetic associations between air pollutants (PM2.5, PM2.5–10, PM10, NO_2_, and NO) and the risk of RA. To ensure the reliability of our findings, we will analyze 2 distinct databases and employ genome-wide association studies (GWAS) to obtain precise genetic information.^[19]^ This genetic-level analysis is expected to provide new insights into the clinical treatment and management of RA, enhancing our understanding of the interplay between environmental factors and autoimmune disorders.

## 2. Study design

### 2.1. Data sources

#### 2.1.1. Exposure data

Our study used various air pollution-related characteristics as exposure indicators, including PM (PM2.5, PM2.5–10, PM10), NO_2_, and NO. The single nucleotide polymorphisms (SNPs) associated with these exposures were selected from the IEU OpenGWAS initiative (https://gwas.mrcieu.ac.uk/datasets/), involving between 423,796 and 456,380 participants of European ancestry (Table 1).

**Table 1:** Characteristics of the GWAS summary datasets for air pollution exposures and RA outcomes, including sample sizes, data sources, and population ancestry.

Phenotypes	ID	PMID	Consortium	Participants
Particulate matter air pollution (PM2.5)	ukb-b-10817	30504882	UKB	423,796
Particulate matter air pollution (PM2.5–10)	ukb-b-12963	30504882	UKB	423,796
Particulate matter air pollution (PM10)	ukb-b-589	30504882	UKB	455,314
Nitrogen dioxide air pollution	ukb-b-2618	30504882	UKB	456,380
Nitrogen oxides air pollution	ukb-b-12417	30504882	UKB	456,380
Rheumatoid arthritis	ebi-a-GCST90018910	34594039	UKB	8255 cases and 409,001 controls
Rheumatoid arthritis	finn-b-M13_RHEUMA	36653562	Finngen	1361 cases and 262,844 controls

GWAS = genome-wide association study, PM = particulate matter, RA = rheumatoid arthritis, UKB = UK Biobank.

#### 2.1.2. Outcome data

The outcome data for RA was obtained from the UK Biobank (UKB) study, which includes 8225 cases and 409,001 controls.^[20]^ Additionally, GWAS data for European RA populations was sourced from the Finngen study, comprising 13,621 cases and 262,844 controls (Table 1). Importantly, there was no sample overlap between the exposure and outcome GWAS.

### 2.2. Study design

**Figure 1.**
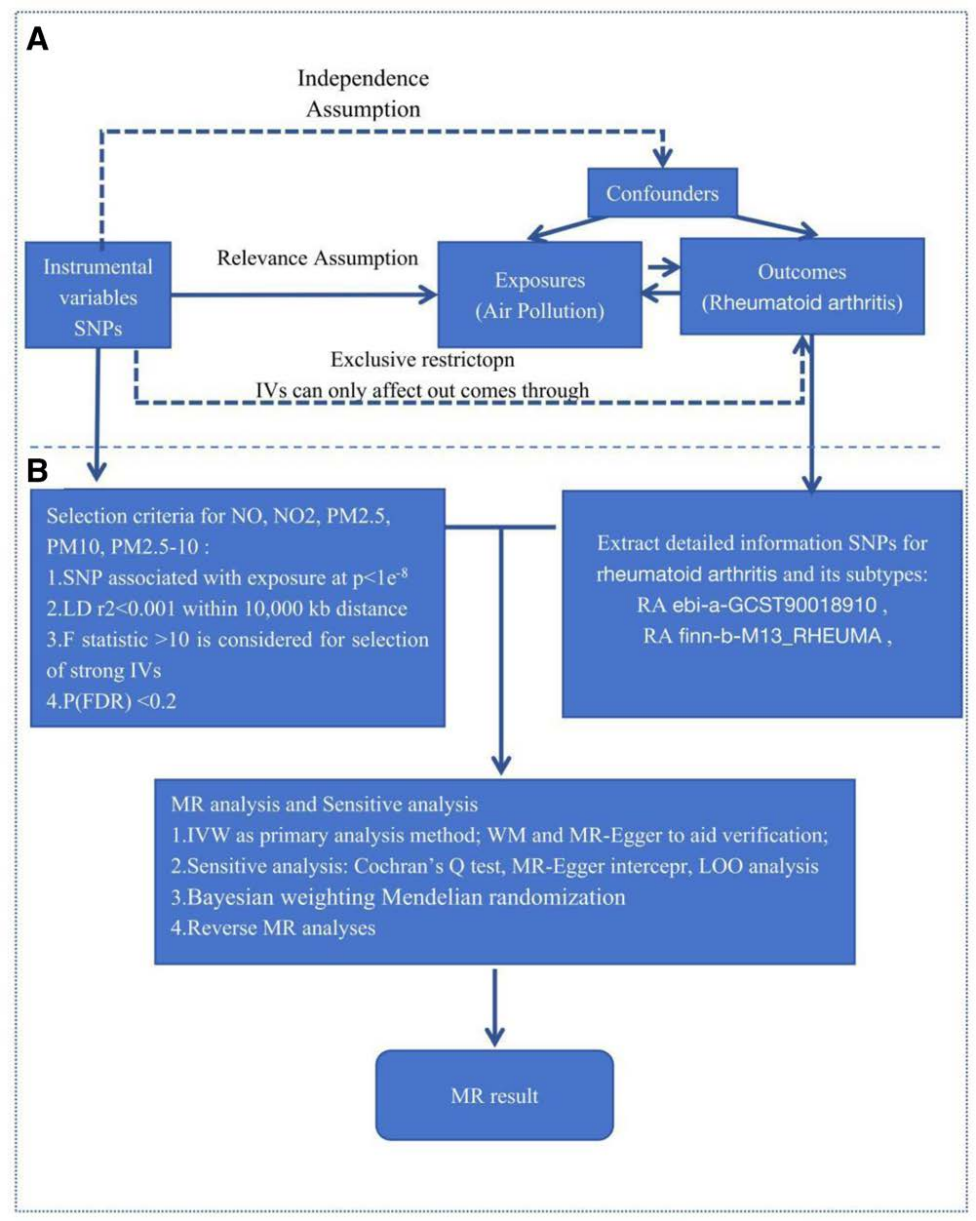
Schematic workflow of Mendelian randomization (MR) analysis for air pollution and rheumatoid arthritis (RA). (A) Assumptions of MR analysis: independence (SNPs as instrumental variables), relevance (strong association between SNPs and exposures), and exclusion restriction (SNPs influence outcomes only through exposures). (B) Selection criteria for instrumental variables (IVs): SNPs associated with air pollutants (NO, NO_2_, PM2.5, PM10, and PM2.5–10) at *P* < 1 × 10^−8^, LD *r*^2^ < 0.001 within 10,000 kb, F-statistic > 10, and FDR correction (*P* < .2). The workflow includes extraction of RA-related SNPs, MR methods (IVW, weighted median, MR-Egger), sensitivity analyses (Cochran Q test, leave-one-out analysis), and reverse MR. FDR = false discovery rate, IVW = inverse variance weighted, PM = particulate matter, SNPs = single nucleotide polymorphisms.

In our study, instrumental variables (IVs) were employed to represent the SNPs.^[21]^ The MR design adheres to 3 key assumptions: (1) IVs must exhibit a strong association with the exposure factors (including PM2.5, PM2.5–10, PM10, NO_2_, and NO); (2) IVs should not be associated with confounding factors; and (3) IVs should influence RA solely through the air pollution factors, with no genetic pleiotropy.^[21,22]^ Our study design is illustrated in a flowchart (Fig. 1). For other European populations concerning PM2.5, PM10, PM2.5–10, NO_2_, and NO, we set significance levels at *P* < 5 × 10⁻⁸, kb > 10,000, and *r*² < 0.001.^[23]^ Additionally, exposure and IVs should have a strong correlation, with an F-statistic >10 serving as a measure of strength; otherwise, they would be considered weak IVs and excluded^[19,24]^ (Table S1, Supplemental Digital Content, https://links.lww.com/MD/P235). To ensure that the causal relationship progresses in the correct direction, we implemented a reverse causation analysis through reverse MR.

### 2.3. MR analysis

Statistical analyses were conducted using R version 4.2.3, and MR analyses were performed using the MendelianRandomization package in R software. We assessed the impact of air pollution-related characteristics on RA by extracting RA data from the UKB and Finnish databases. We used inverse variance weighting (IVW) as our primary analytical method. IVW integrates the Wald values of each SNP through a meta-analytic approach, providing a comprehensive estimate of the exposure’s effect on the outcome. When assuming all SNPs are valid instruments, IVW provides the most precise estimate of causal effects. To further enhance the reliability of our findings, we conducted additional analyses using MR-Egger, weighted median (WM), and weighted mode methods. The MR-Egger regression analysis demonstrated robustness against invalid instruments and addressed potential biases by introducing parameters to tackle horizontal pleiotropy issues. The WM method extracts up to 50% of the analytical information from genetic variation associated with zero IV.^[25–27]^

To validate the reliability and strength of our primary findings, we performed extensive sensitivity analyses: (1) a *P*-value below .05 for the MR-Egger intercept indicates the presence of horizontal pleiotropy^[27,28]^; (2) we applied Cochran Q test to detect heterogeneity arising from horizontal pleiotropy and other biases, with a *P*-value below .05 suggesting heterogeneity. To address this heterogeneity, the IVW method employs a random effects model, while the IVW fixed effects model serves as an alternative^[29]^; (3) we conducted a leave-one-out analysis to evaluate the stability of effect sizes and identify SNPs that significantly influence the associations. This involved systematically removing 1 SNP at a time and applying the IVW method to the remaining SNPs^[27]^; (4) for positive results, we performed false discovery rate (FDR) testing^[30]^ and Bayesian weighted Mendelian randomization (BWMR) analysis,^[31]^ considering results reliable when *P *< .05.

## 3. The results of MR

**Figure 2.**
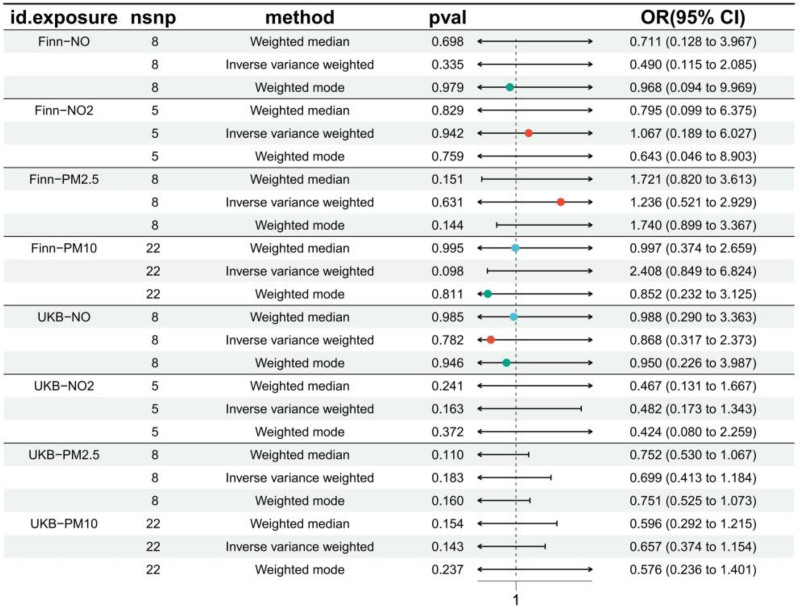
Causal associations between air pollution and rheumatoid arthritis risk in Finnish and UK Biobank (UKB) cohorts. Results from forward Mendelian randomization analysis using inverse variance weighted (IVW), weighted median (WM), and weighted mode methods. Odds ratios (OR) and 95% confidence intervals (CI) are shown for exposures: NO, NO_2_, PM2.5, and PM10. Key findings: no significant causal associations observed for PM2.5 (Finn: OR = 1.236, *P* = .631; UKB: OR = 0.699, *P* = .183), PM10 (Finn: OR = 2.408, *P* = .098; UKB: OR = 0.657, *P* = .143), or gaseous pollutants (all *P* > .05). PM = particulate matter.

Our study strictly adhered to the 3 core assumptions of MR (Fig. 1). We extracted air pollution data comprising 5 to 22 SNPs from the IEU Open GWAS project for subsequent analyses; however, we were unable to extract data for PM2.5–10. The F-statistic values ranged from 29.97 to 108.96, indicating the absence of weak IVs (Table S1, Supplemental Digital Content, https://links.lww.com/MD/P235). Given that IVW provides the most accurate estimates of causal effects, we primarily evaluated significant genetically predicted causal relationships based on IVW *P*-values. In both databases, we did not observe a significant genetically predicted causal relationship between air pollution and RA (Table S2, Supplemental Digital Content, https://links.lww.com/MD/P235), and the main analysis results are illustrated using a forest plot (Fig. 2).

For the UK Biobank (UKB) data, the results were as follows: PM2.5 (*P* = .183; odds ratio [OR]: 0.71; 95% confidence interval [CI]: 0.27–1.91), PM10 (*P* = .143; OR: 0.657; 95% CI: 0.374–1.154), NO₂ (*P* = .163; OR: 0.482; 95% CI: 0.173–1.343), and NO (*P* = .782; OR: 0.868; 95% CI: 0.317–2.373). For the Finnish data, the results were: PM2.5 (*P* = .631; OR: 1.236; 95% CI: 0.521–2.929), PM10 (*P* = .098; OR: 2.408; 95% CI: 0.849–6.824), NO₂ (*P* = .163; OR: 0.482; 95% CI: 0.173–1.343), and NO (*P* = .782; OR: 0.868; 95% CI: 0.317–2.373). Additionally, the MR-Egger, WM, and weighted mode methods did not reveal any significant causal relationships.

## 4. Heterogeneity and sensitivity analysis

We conducted heterogeneity tests and sensitivity analyses to ensure the robustness of our results. Cochran Q test indicated no heterogeneity in the main results (*P* > .05), and the MR-Egger intercept showed no signs of potential horizontal pleiotropy (*P* > .05) (Tables S3 and S4, Supplemental Digital Content, https://links.lww.com/MD/P235). Furthermore, we performed reverse MR analyses and confirmed the stability of the results through leave-one-out testing. Figures S1–S16, Supplemental Digital Content, https://links.lww.com/MD/P236 displays the leave-one-out testing and scatter plots for the MR analyses conducted in both databases; detailed visualizations of sensitivity analyses, including scatter plots illustrating genetic correlations between each air pollutant (including PM2.5, PM10, nitrogen dioxide, and nitrogen oxides) and RA under different MR methods, and leave-one-out forest plots for all instrumental SNPs, are provided in Figures S1–S16, Supplemental Digital Content, https://links.lww.com/MD/P236.

## 5. Reverse MR

**Figure 3.**
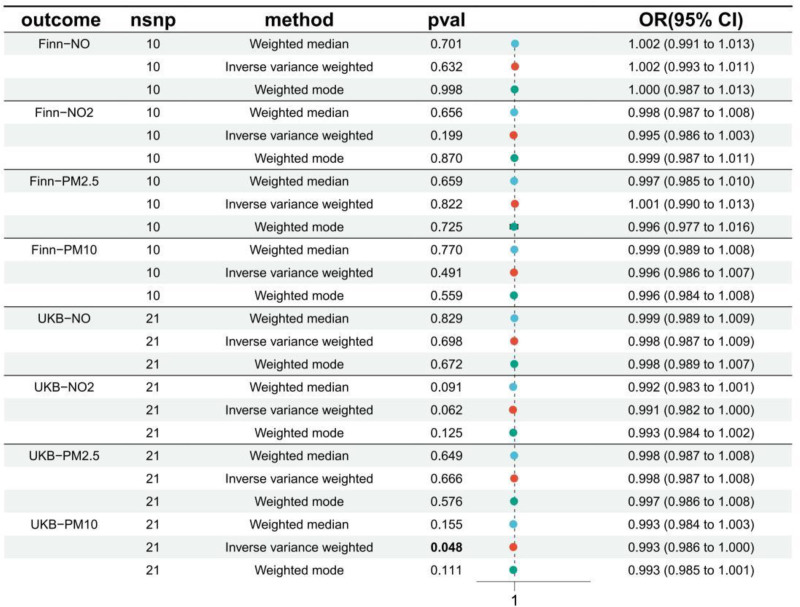
Reverse Mendelian randomization (MR) analysis of rheumatoid arthritis on air pollution exposure. Results from reverse MR assessing potential causal effects of RA on air pollution (NO, NO_2_, PM2.5, PM10). Analyses used IVW, weighted median, and weighted mode methods. No significant associations were observed for most pollutants (all *P* > .05), except for UKB–PM10 (IVW: OR = 0.993, *P* = .048), though FDR correction and Bayesian weighted MR suggested non-significance (*P* = .075). FDR = false discovery rate, IVW = inverse variance weighted, PM = particulate matter.

In the reverse MR analysis (Fig. 3 and Table S5, Supplemental Digital Content, https://links.lww.com/MD/P235), we found that PM10 had a *P*-value of .048 (OR: 0.993; 95% CI: 0.986–1.000). To further validate the reliability of the results, we conducted FDR correction, yielding *P*(FDR) = .048, and the BWMR value was 0.075. Therefore, we do not consider a correlation between RA and PM10.

## 6. Discussion

RA is a complex autoimmune disorder that poses significant challenges for early diagnosis and effective treatment, affecting approximately 1% of the global population, with higher prevalence rates among middle-aged and older women.^[32]^ Beyond joint symptoms, RA can present with extra-articular manifestations that impact the skin, mucous membranes, cardiovascular system, and lungs, severely disrupting daily life and work activities.^[33,34]^ Traditional observational studies face numerous challenges in elucidating the mechanisms underlying RA, particularly due to confounding factors such as dietary habits,^[35]^ body mass index,^[36]^ smoking, and alcohol consumption.^[37]^ These variables not only influence metabolite levels but may also significantly affect the disease trajectory. Therefore, it is crucial to investigate the etiological mechanisms of RA, reduce risk factors, and explore preventive measures. Recent research has increasingly focused on the potential causal relationship between air pollution and RA, especially through genetic prediction, which has emerged as a prominent area of study. This perspective offers valuable insights for formulating public health policies, emphasizing how environmental factors, particularly air pollution, influence RA risk through genetic susceptibility. Such understanding can aid in developing more effective prevention and intervention strategies, ultimately improving patients’ quality of life.

Recent studies on the relationship between air pollution and RA have been increasing, revealing significant associations between exposure to PM2.5, NO_2_, and other pollutants and the incidence of RA and related biomarkers. Evidence suggests that air pollution may heighten susceptibility to RA and exacerbate its severity by inducing inflammatory mediators and autoantibody production.^[38]^ Notably, some studies have identified significant correlations between PM2.5 and NO_2_ exposure and increased readmission rates among RA patients, particularly among women and the elderly.^[6]^ A retrospective study by Giovanni Adami et al established a close correlation between long-term air pollution exposure and heightened risk of autoimmune diseases, including RA.^[39]^ Additionally, a longitudinal study involving 342,973 participants demonstrated that prolonged exposure to air pollution significantly elevates RA risk, particularly among genetically susceptible individuals.^[40]^ However, the interplay between environmental triggers and immune dysregulation extends beyond pollutants. For instance, emerging evidence suggests that environmental allergens, such as pollen, may exacerbate systemic inflammation through neuro-immune interactions, potentially influencing autoimmune pathways.^[41]^ Di Gioacchino et al highlighted that pollen allergy could modulate central nervous system responses, thereby indirectly affecting immune homeostasis and autoimmune disease progression.^[41]^ This underscores the complexity of environmental exposures in RA pathogenesis, where multiple factors (ranging from PM to allergens) may synergistically contribute to disease susceptibility.

Despite efforts to mitigate confounding effects, inherent limitations in retrospective studies persist. Several meta-analyses have failed to confirm a negative correlation between PM exposure and RA incidence, especially in the Stockholm region, where findings are inconsistent.^[42,43]^ An experimental study assessing PM exposure in RA-related autoantibody carriers revealed no significant association between autoantibodies and surrounding PM concentrations.^[44]^ Most epidemiological studies employ case-control designs, complicating the determination of causal relationships; the temporal sequences involved can also be ambiguous, potentially resulting in uncontrolled confounding factors. Even prospective observational studies encounter challenges such as visit bias and inadequate sample sizes.^[45,46]^ Thus, reliance solely on observational studies is insufficient to definitively establish whether air pollution directly causes RA. Furthermore, psychological factors, often overlooked in mechanistic studies, play a critical role in RA progression. Chronic pain, disability, and the unpredictability of disease flares can lead to anxiety, depression, and reduced quality of life, creating a bidirectional relationship between psychological stress and inflammatory activity.^[47]^ Frydas et al emphasized that psychological distress in RA patients may amplify systemic inflammation through hypothalamic–pituitary–adrenal axis dysregulation, thereby exacerbating joint damage and autoantibody production.^[47]^ This highlights the need for a holistic approach to RA management, integrating environmental, genetic, and psychosocial dimensions.

Among the examined studies, 2 cohort studies failed to find significant associations between PM2.5 exposure and rheumatoid factor or anti-citrullinated protein antibodies (ACPA) in at-risk populations.^[43,44]^ Conversely, several cross-sectional studies have reported a positive correlation between PM2.5 and ACPA. For instance, a study involving over 500 confirmed RA American veterans indicated a linear relationship between PM2.5 exposure and ACPA levels; however, no correlation was found between PM2.5 and RA itself.^[48]^ Bernatsky et al found that for every additional 10 tons of industrial PM2.5 exposure within 2.5 kilometers of a pollution source, the probability of ACPA positivity increased by 2%.^[49]^ Regarding PM10, 4 studies investigated its exposure in relation to RA biomarkers.^[43,44,48]^ Utilizing location-based emission data and/or modeling to measure RF and ACPA, these studies did not find significant associations with any biomarkers.

In our study, by leveraging the latest GWAS data, we aimed to overcome biases associated with traditional observational studies.^[40]^ While randomized controlled trials are considered the gold standard for causal research, their high costs and feasibility issues render MR an effective alternative. MR studies simulate random assignment to some extent, effectively mitigating confounding biases that arise from the random allocation of SNPs.^[18]^ Our findings did not provide evidence of a direct genetically predicted causal relationship between the 4 phenotypes of air pollution (PM2.5, PM10, NO_2_, and NO) and RA risk. This suggests that while existing studies show significant associations between air pollution and RA, the influence may be mediated indirectly by various factors. For instance, gaseous pollutants may absorb Ultraviolet B radiation and reduce serum vitamin D levels, potentially increasing RA incidence.^[11]^ This indicates that the etiology of RA involves a complex interplay of multiple factors, including environmental triggers, genetic predisposition, and psychosocial stressors.

Despite our thorough investigation, we acknowledge several limitations in this study. First, due to constraints within the current GWAS database, we obtained only a limited number of IVs and were able to extract only 2 strongly correlated IVs. As alternative analytical methods, apart from IVW, were unsuitable, we could not identify potential heterogeneity and pleiotropy through sensitivity analyses. However, we conducted FDR correction on positive results and subsequently performed BWMR analysis to ensure the accuracy of our findings. Second, our study was limited to European participants, which restricts the generalizability of our results. To enhance accuracy, we analyzed data from 2 databases and applied FDR correction and BWMR analysis to positive results. Additionally, the heterogeneity among RA patients should be considered, as different causal relationships may exist between RA and air pollution. Future studies should incorporate diverse populations and multi-omics data to disentangle the roles of environmental, genetic, and psychosocial factors in RA pathogenesis.

## 7. Conclusion

Our study results indicate that there is no significant causal relationship between genetically predicted air pollutants and the incidence of RA in the European population. To validate these findings, future research will utilize the latest and more comprehensive GWAS data to further explore the impact of air pollution on RA. Such future investigations will enhance our understanding of RA.

## Acknowledgments

This study was supported by publicly available genome-wide association studies (GWAS), including the GWAS Catalog.

## Author contributions

**Conceptualization:** Chengrui Yang, Zhiguo Du, Jing Ma.

**Funding acquisition:** Chengrui Yang.

**Investigation:** Chengrui Yang, Zhiguo Du, Jing Ma.

**Methodology:** Chengrui Yang, Zhiguo Du, Jing Ma.

**Project administration:** Jianyong Zhao.

**Resources:** Lele Guo.

**Software:** Chengrui Yang.

**Supervision:** Jianyong Zhao.

**Validation:** Zhiguo Du, Huidong Zhang, Yu Tian.

**Visualization:** Jing Ma.

**Writing – original draft:** Chengrui Yang, Zhiguo Du, Jing Ma.

**Writing – review & editing:** Lele Guo, Huidong Zhang, Yu Tian, Jianyong Zhao.

## Supplementary Material


