# Supplementary material for: Causal relationship between air pollution and rheumatoid arthritis: A two-sample Mendelian randomization study
Source: Medicine (Baltimore). 2025 Jun 20;104(25):e42901. doi: 10.1097/MD.0000000000042901 (PMC12187290; doi:10.1097/MD.0000000000042901)
Supplement: Supplementary file 2 [file medi-104-e42901-s002.docx]

Supplementary Figure 1. Mendelian randomization results of NO and RA: Scatter plot of genetic correlations between NO and RA using different MR methods,The slopes of the lines represent the causal effects of each method. （Finngen study）

Supplementary Figure 2. Mendelian randomization results of NO and RA: Forest plot of the leave-one-out analysis for SNPs related to NO and RA.（Finngen study）

Supplementary Figure 3. Mendelian randomization results of NO2 and RA: Scatter plot of genetic correlations between NO2 and RA using different MR methods, The slopes of the lines represent the causal effects of each method. (Finngen study)

Supplementary Figure 4. Mendelian randomization results of NO2 and RA: Forest plot of the leave-one-out analysis for SNPs related to NO2 and RA.(Finngen study)

Supplementary Figure 5.Mendelian randomization results of PM2.5 and RA: Scatter plot of genetic correlations between PM2.5 and RA using different MR methods. The slopes of the lines represent the causal effects of each method.(Finngen study)

Supplementary Figure 6. Mendelian randomization results of PM2.5 and RA: Forest plot of the leave-one-out analysis for SNPs related to PM2.5 and RA.(Finngen study)

Supplementary Figure 7. Mendelian randomization results of PM10 and RA: Scatter plot of genetic correlations between PM10 and RA using different MR methods. The slopes of the lines represent the causal effects of each method.(Finngen study)

Supplementary Figure 8. Mendelian randomization results of PM10 and RA: Forest plot of the leave-one-out analysis for SNPs related to PM10 and RA.(Finngen study)

Supplementary Figure 9. Mendelian randomization results of NO and RA: Scatter plot of genetic correlations between NO and RA using different MR methods. The slopes of the lines represent the causal effects of each method.(UK Biobank study)

Supplementary Figure 10. Mendelian randomization results of NO and RA: Forest plot of the leave-one-out analysis for SNPs related to NO and RA.(UK Biobank study)

Supplementary Figure 11. Mendelian randomization results of NO2 and RA: Scatter plot of genetic correlations between NO2 and RA using different MR methods. The slopes of the lines represent the causal effects of each method.(UK Biobank study)

Supplementary Figure 12. Mendelian randomization results of NO2 and RA: Forest plot of the leave-one-out analysis for SNPs related to NO2 and RA.(UK Biobank study)

Supplementary Figure 13.Mendelian randomization results of PM2.5 and RA: Scatter plot of genetic correlations between PM2.5 and RA using different MR methods. The slopes of the lines represent the causal effects of each method.(UK Biobank study)

Supplementary Figure 14. Mendelian randomization results of PM2.5 and RA: Forest plot of the leave-one-out analysis for SNPs related to PM2.5 and RA.(UK Biobank study)

Supplementary Figure 15. Mendelian randomization results of PM10 and RA: Scatter plot of genetic correlations between PM10 and RA using different MR methods. The slopes of the lines represent the causal effects of each method.(UK Biobank study)

Supplementary Figure 16. Mendelian randomization results of PM10 and RA: Forest plot of the leave-one-out analysis for SNPs related to PM10 and RA.(UK Biobank study)
